# OGM and WES identifies translocation breakpoints in PKD1 gene in an polycystic kidney patient and healthy baby delivered using PGT

**DOI:** 10.1186/s12920-023-01725-2

**Published:** 2023-11-13

**Authors:** Peiwen Xu, Lijuan Wang, Jing Li, Sexin Huang, Ming Gao, Ranran Kang, Jie Li, Hongqiang Xie, Xiaowei Liu, Junhao Yan, Xuan Gao, Yuan Gao

**Affiliations:** 1 Center for Reproductive Medicine, Cheeloo College of Medicine, Shandong University, Jinan, 250012 Shandong China; 2 Shandong Key Laboratory of Reproductive Medicine, Shandong Provincial Hospital Affiliated to Shandong First Medical University, Jinan, China; 3 National Research Center for Assisted Reproductive Technology and Reproductive Genetics, Shandong University, Jinan, 250012 Shandong China; 4 Key laboratory of Reproductive Endocrinology of Ministry of Education, Shandong University, Jinan, 250012 Shandong China; 5 Shandong Provincial Clinical Medicine Research Center for Reproductive Health, Shandong University, Jinan, 250012 Shandong China

**Keywords:** ADPKD, PKD1, WES, Karyotype analysis, Reciprocal translocations, PGT

## Abstract

**Background:**

Autosomal dominant polycystic kidney disease (ADPKD) is one of the most common autosomal dominant genetic diseases. Whole exome sequencing (WES) is a routine tool for diagnostic confirmation of genetic diseases, and it is usually performed to confirm the clinical diagnosis in ADPKD. Reciprocal translocation is the most common chromosomal structural abnormalities and most of its carriers have normal phenotypes until they are encountered infertility problems in adulthood. However, for the polycystic kidney disease caused by abnormal chromosome structure, WES is difficult to achieve the purpose of gene diagnosis.

**Methods:**

ADPKD-related genes were detected by WES; Chromosomal karyotyping and Optical Genome Mapping (OGM) were used to detect structural variant; The genomic break-point locations and the abnormal splicing were detected by reverse transcription-PCR and Sanger sequencing; The karyomapping gene chip and Next-Generation Sequencing (NGS) were performed to screen aneuploidy and to distinguish the non-carrier embryos from the carrier embryos.

**Results:**

No pathogenic variant was found after the first round of WES analysis. Karyotyping data showed 46, XX, t (16; 17) (p13.3; q21.3). With the help of OGM, the translocation breakpoint on chromosome 16 was located within the PKD1 gene. With re-analysis of WES raw data, the breakpoint of translocation was verified to be located at the c.10618 + 3 of PKD1 gene. Based on this molecular diagnosis, a non-carrier embryo was selected out from three blastocysts. With preimplantation genetic testing (PGT) after in vitro fertilization (IVF), it was then transferred into uterus. With confirmation by prenatal and postnatal testing, the pedigree delivered a healthy baby.

**Conclusion:**

We identified a case of ADPKD caused by balanced translocation and assisted the patient to have a healthy child. When the phenotype was closely related with a monogenic disease and the WES analysis was negative, chromosomal structural analysis would be recommended for further genetic diagnosis. Based on the precision diagnosis, preventing the recurrence of hereditary diseases in offspring would be reachable.

## Introduction

Reciprocal translocation is a common chromosomal abnormality, occurring in 1/500 to 1/625 human newborns [1]. While carriers of balanced reciprocal translocations typically do not exhibit any observable phenotypes, meiosis in germ cells with balanced translocations can lead to infertility or production of unbalanced gametes, which can result in miscarriage and unbalanced progeny [2]. Additionally, approximately 6% of reciprocal carriers may experience symptoms such as autism, intellectual disabilities, or congenital abnormalities due to the disruption of gene structure caused by the breakpoints of translocation located inside the gene [3, 4].

Chromosomal rearrangements can be detected through various methods such as G-band karyotyping, Chromosome microarray (CMA), FISH, low-depth whole-genome sequencing (WGS), long-read sequencing technologies, and Optical genome mapping (OGM). However, karyotyping has a low resolution of approximately 5–10 Mb on average. CMA is unable to detect the mosaicism lower than 5–20% or balanced chromosomal aberrations [5]. FISH has limitations such as the need for prior knowledge of loci and the use of specific fluorescent probes. Sequencing-based detection of some Structure Variantions (SVs) is challenging due to the relatively limited read length and the repetitive nature of sequences at some SV breakpoints, which are often mediated by non-allelic homologous recombination of repeats [6]. Long-read sequencing technologies may generate high assembly error rates and can be cost-prohibitive [7]. However, OGM is a promising non-sequencing genome imaging tool that can detect copy number and structural variants at high resolution [6], making it a key genomic technology for detecting all classes of SVs in many disorders.

ADPKD is a common monogenic disease, affecting between one in 1000 and one in 2500 individuals [8, 9]. It is caused by mutations in *PKD1* or *PKD2,* which account for approximately 78 and 15% of all ADPKD cases, respectively [10]. Currently, Next-Generation Sequencing (NGS) is the primary molecular detection method for ADPKD-related pathogenic gene exons testing, with a diagnostic rate of 80–90% [11, 12]. Despite the high success rate of these laboratory procedures, around 10.1% of clinically diagnosed cases do not receive molecular confirmation [12]. This may be due to tissue mosaicisms or different mutational events, such as structural rearrangements that cannot be resolved by standard techniques. According to the ADPKD mutation database, large genomic rearrangements are rare, accounting for less than 4% of pathogenic mutations in the PKD1 gene [12].

Here we report a case of ADPKD caused by structure rearrangement, whose whole exome sequencing(WES) was negative for pathogenic variants. Based on karyotype and OGM analyses, the breakpoint of a causal SV in the PKD1 gene was accurately identified. Preimplantation genetic testing (PGT) was performed after IVF for blastocysts screening, and a noncarrier euploidy embryo was selected out for transferring by karyomap gene chip and NGS. Eventually, a healthy baby was delivered after further prenatal diagnosis and postnatal diagnosis.

## Materials and methods

### Patients

**Fig. 1:**
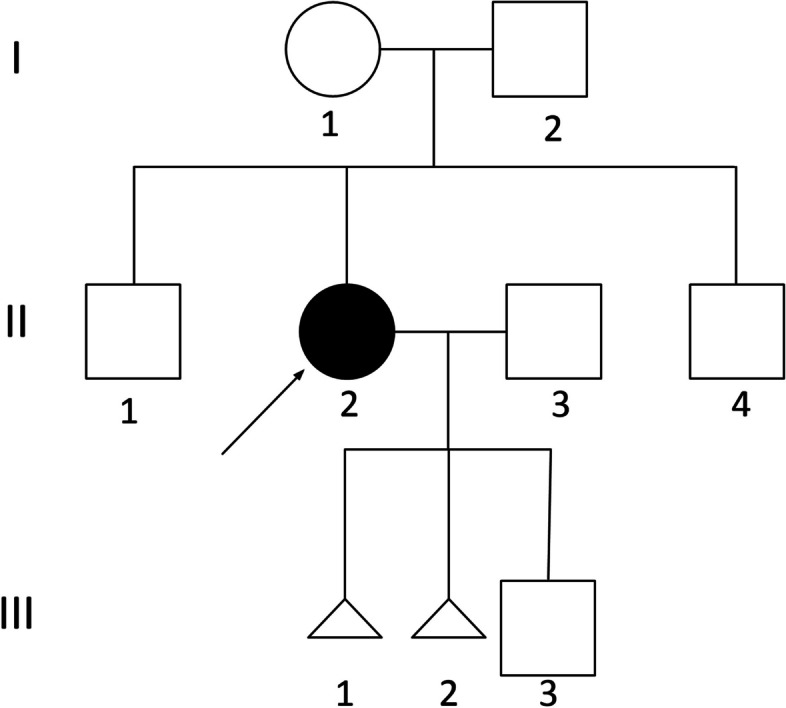
Pedigree of the proband’s family. Arrows indicate the probands. Squares: males; circles: females; triangles: miscarried embryos

A Chinese family suffered from ADPKD and a history of spontaneous abortions (Fig. 1). The patient came to the reproductive medicine center for fertility problems. Blood and biochemical detection showed no abnormality in her husband.

### Whole exome sequencing

Following the manufacturer’s instructions, genomic DNA was extracted from peripheral blood lymphocytes using commercially available kits (ZEESAN). The whole exon was sequenced using the Illumina platform NovaSeq 6000 sequencer (Illumina, USA) and IDT capture kit IDT xGen Exome Research Panel (IDT, USA). Sequence reads were aligned to the human reference genome (UCSC HG19) by the BWA algorithm, and the instruments were run at default settings [13]. Data were annotated by the literature reporting method [14]. After the screening, variants were predicted by bioinformatics software (PolyPhen2, LRT, Mutation Taster, etc.). We focused on the analysis of genes related to polycystic kidney disease to obtain suspected candidate variants.

### G-band karyotype

The preparation of the peripheral blood sample was done by culturing, harvesting, and metaphase preparation as previously described [6]. At least 25 mitoses were analyzed (median number of analyzed mitoses in this material, 25). Nomenclature went according to the International System for Human Cytogenetic Nomenclature 2016 (ISCN, 2016).

### Optical genome mapping (OGM) and structural variant calling

Same as previously reported, high molecular weight (HMW) genomic DNA was extracted from fresh blood collected in EDTA tubes using the Bionano Prep Blood and Cell Culture DNA Isolation Kit (Bionano Genomics). DNA was quantified using Qubit 3.0 Fluorometer (Thermo Fisher Scientific, USA). DNA labeling was carried out according to Bionano Prep DLS Labeling Kit Protocol (Bionano Genomics). The DNA labeled with markers was loaded into the flowcell of the Saphyr Chip (Bionano Genomics), which was then run to achieve a throughput of 800Gb [15].

Annotated de novo assembly was executed on Bionano Solve software V3.7. Data visualization of structural variants was performed on Bionano Access V1.7.

### Breakpoint verification

We designed PCR primers to detect the translocation breakpoints in the sample using Primer3 (https://primer3.ut.ee/). The sequences of all primers used in this study are provided in Table 1. PCR was carried out using Taq polymerase (R500Z, TaKaRa), and the resulting products were electrophoresed on a 2.0% agarose gel and sequenced using Sanger sequencing on an ABI3730XL sequencer (Applied Biosystems) [16].

**Table 1:** Design of PCR primers to validate translocations

Breakpoint	Primer	Primer Sequence
chr16	PKD1–16-17-AF	atggtgtggctgctatggaa
	PKD1–16-17-AR	ttcacagcctaccatgtccc
chr17	PKD1–17-16-AF	accaacacaccggctaatgt
	PKD1–17-16-AR	gggaagcagagacagacctg

### Reverse transcription-PCR

Total RNA was extracted from the peripheral blood according to the protocol of the total RNA extraction reagent kit (Takara). Complementary DNA was synthesized according to the protocol of PrimeScript™ II 1st Strand cDNA Synthesis Kit (Takara). Specific primers were designed for the mRNA sequence of the mutated PKD1 gene. The forward primer of mutated *PKD1* is 5′- cctacccaagacacccacat-3′, and the reverse primer is 5′-ttcaccgtgttagccaggat-3′, with a 216 bp PCR product. And the forward primer of normal *PKD1* is 5′- cctacccaagacacccacat-3′, and the reverse primer is 5′-gcatgccatgtagcctcttg-3′, with a 555 bp PCR product.

### IVF-TE biopsy and whole genome amplification

Informed consent was signed for the PGT cycle. The standard techniques were used for IVF. Briefly, retrieved MII oocytes were produced by using intracytoplasmic sperm injection, and then were cultured to develop to the blastocyst stage. The criteria for grading blastocyst were following the recommendation by Schoolcraft et al. Totally 3 embryos were biopsied after IVF on day 5 or 6 and biopsied cells were washed and transferred to a PCR tube containing 2.5 μL phosphate buffer saline (PBS) under strictly sterile and DNA-free conditions against contamination. Whole-genome amplification (WGA) was performed following the protocol of the REPLI-g Single Cell Kit (QIAGEN GmbH, Hilden, Germany) [16, 17]. The WGA product was used for the subsequent tests, including *PKD1* mutation analysis, aneuploidy analysis, and reciprocal translocation carrier screening.

### Haplotype analysis, testing for aneuploidy, and chromosome rearrangement

As described previously, the WGA products and blood DNA samples from family members were analyzed using SNP-array analysis according to the manufacturer’s instructions. The data obtained were scanned using an iScan Bead Array Reader (Illumina, San Diego, CA, USA) and SNP calling was carried out to identify informative SNPs. To prevent any misinterpretation that might arise from potential recombination events during meiosis, we chose to analyze regions that were within 2 M bp of the breakpoints [18]. To detect aneuploidy or CNVs, we processed the microarray scanning results using the B allele frequency and Log R ratio. The core algorithm we used was based on cnvPartition, as previously reported [19, 20]. For chromosome rearrangements, we utilized the molecular karyotype of an unbalanced embryo to identify the estimated positions of the breakpoints. We then analyzed informative SNPs within a 2 Mb region surrounding the breakpoints to establish haplotypes [18].

### Prenatal and postnatal testing

Intrauterine gestation was confirmed by ultrasound examination after the embryo transference and the karyotype of transferred embryo was verified with amniotic fluid cells in the second trimester and umbilical cord blood at birth, respectively.

## Results

### Medical history of the patient in this family

**Fig. 2:**
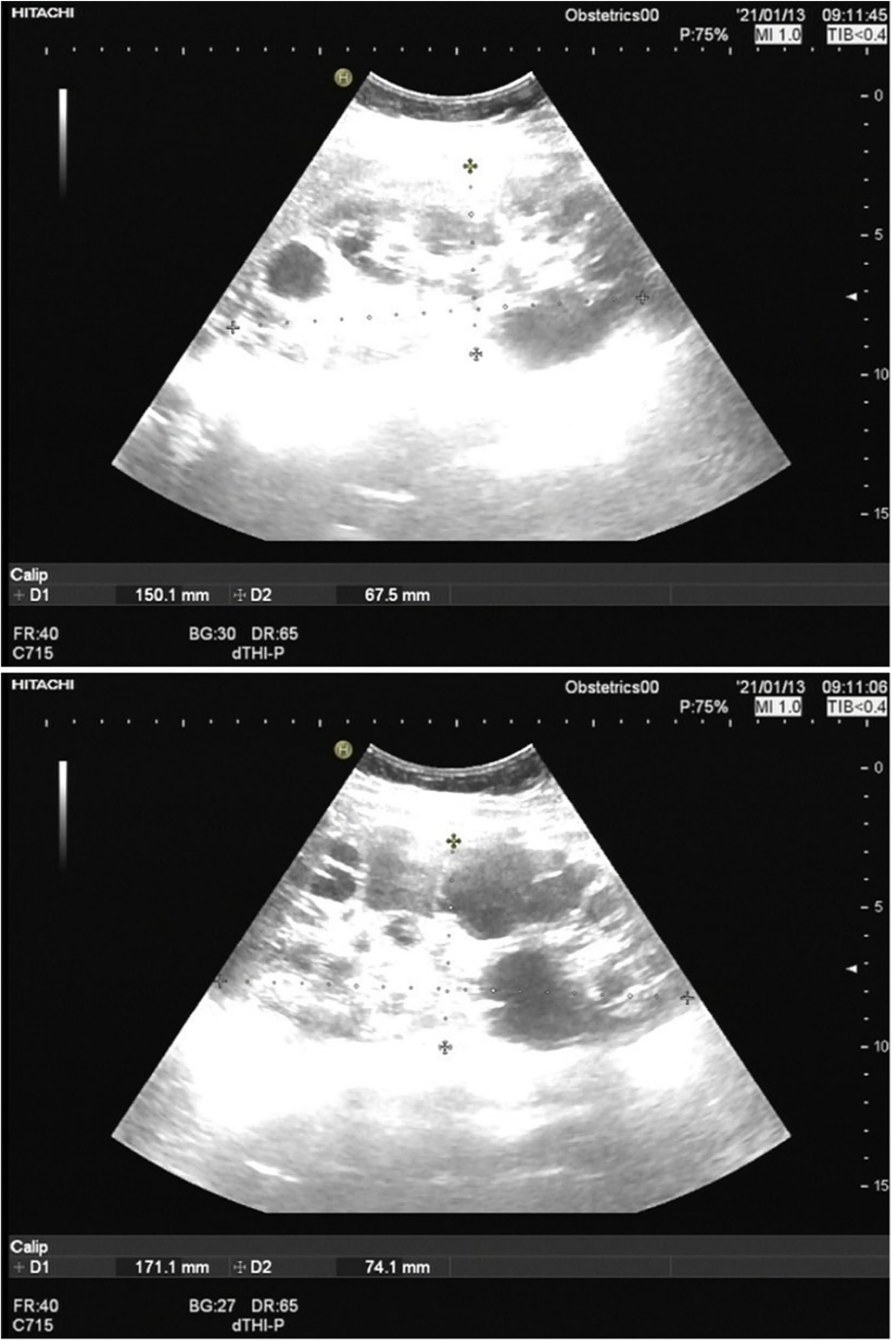
The bilateral renal cysts of II 2. **a** Left kidney, **b** Right kidney. At 34 years of age, the left kidney measured 15.0× 6.8 cm. The right kidney measured 17.1× 7.4 cm. Both kidneys were larger than normal female kidneys of the same age. The plus areas(+) represent the length and width of the kidney. Vesicle-like echoes of multiple sizes were observed in both sides of the renal parenchyma. The largest cyst size measured 3.4 × 3.1 cm in the left kidney and 4.1 × 3.6 cm in the right kidney

The proband was a 35-year-old woman with hepatic cysts and bilateral renal cysts (Fig. 2), whose urinary occult blood was positive, blood pressure (systolic and diastolic pressure) was normal, serum creatinine was normal. At the age of 32, she had a biochemical pregnancy miscarriage. At 33, she got pregnant again and the pregnancy was selectively terminated at the 8th week due to absence of fetal heartbeat (Fig. 1).

### A pathogenic variant was found in the de novo reciprocal translocation family

**Fig. 3:**
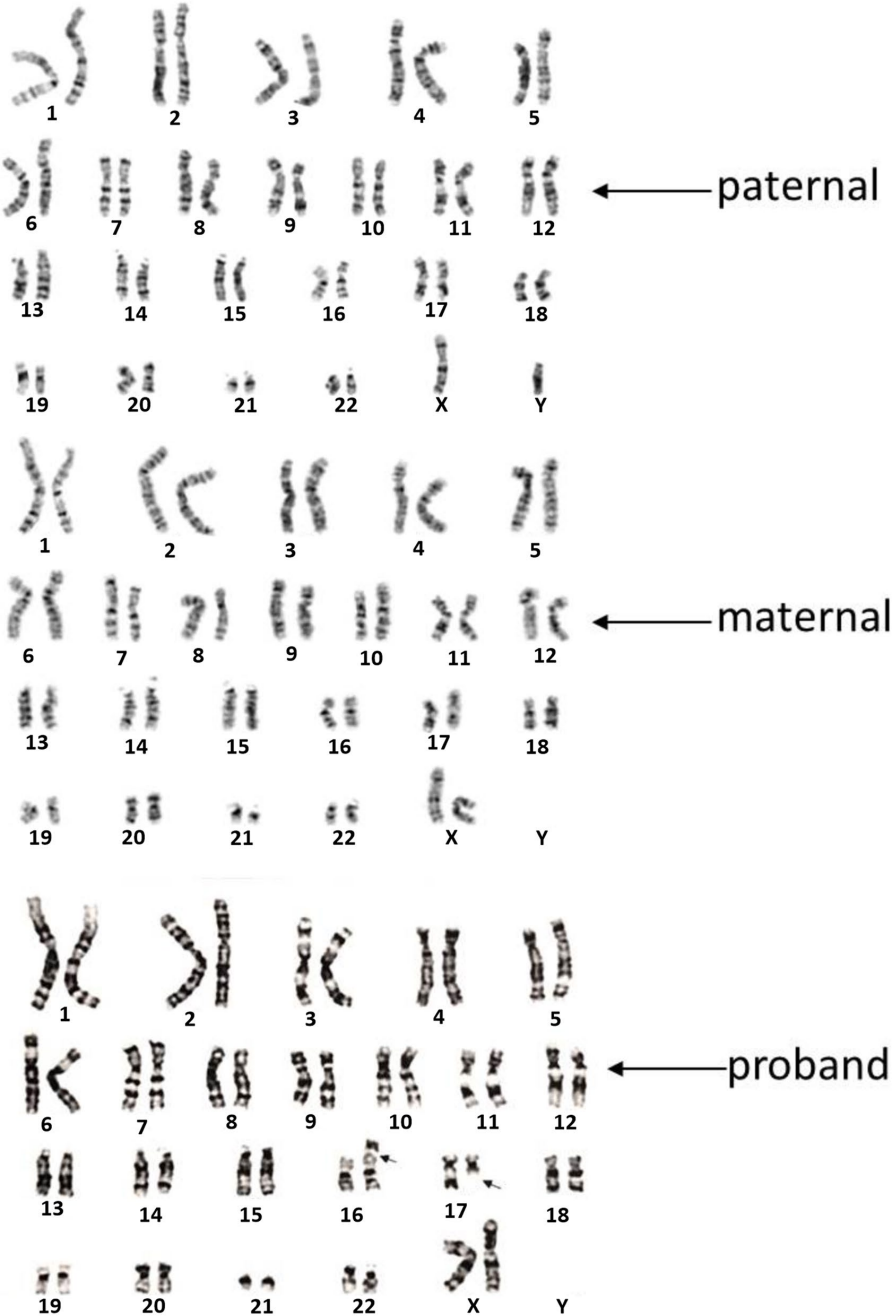
G-band karyotype of the pedigree. The reciprocal translocation between chromosomes 16 and 17 of the proband is illustrated

**Fig. 4:**
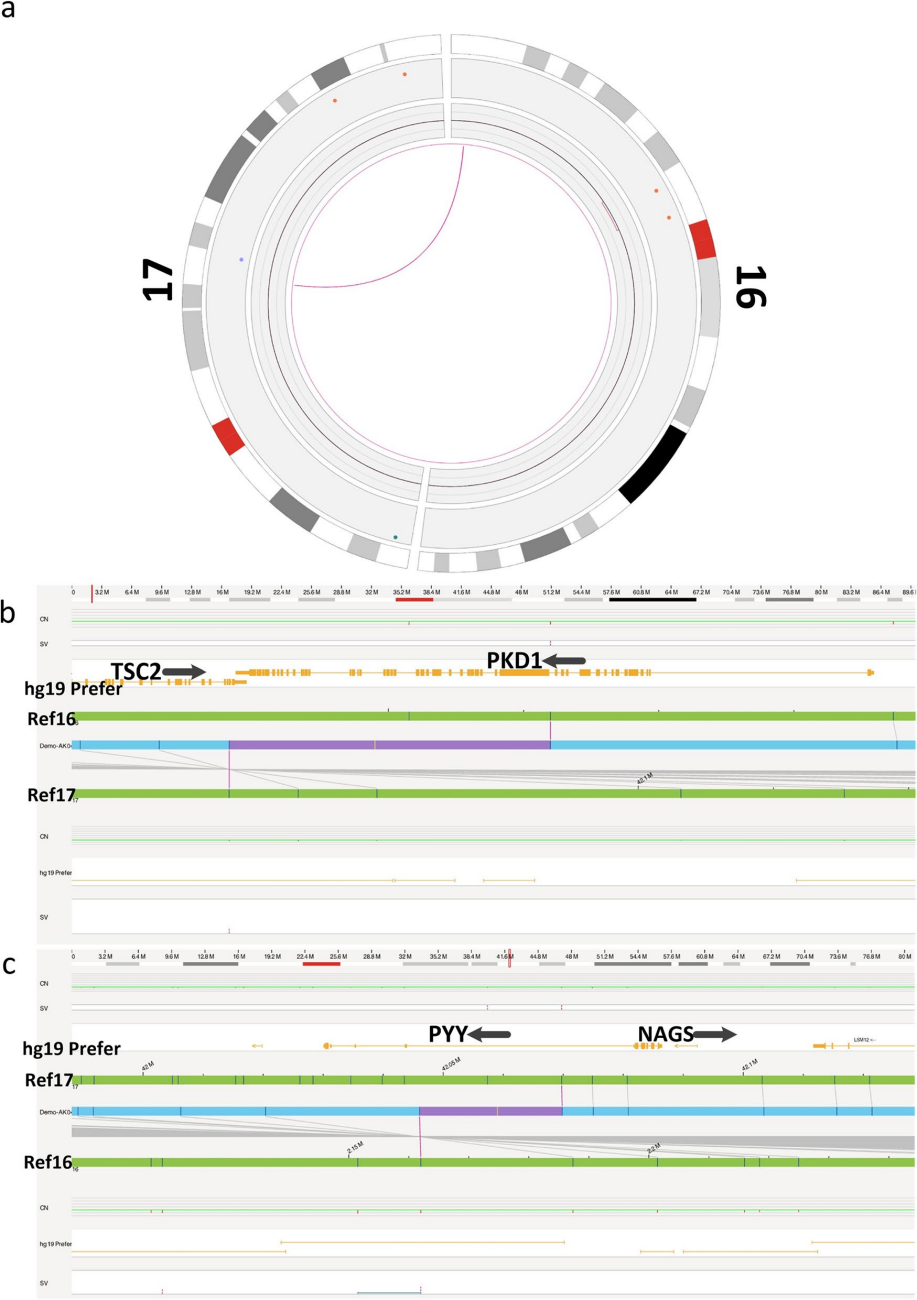
Characterization of disease-causing variants at the *PKD1* locus. **a** Optical genome mapping (OGM) shows the translocation between chromosomes 16 and 17(GRCh37). **b** The breakpoint of the chr16 is situated between *PKD1* gene intron 14 and exons 46, thereby disrupting the gene. **c** The breakpoint of the chr17 is situated *PYY* gene intron 1(GRCh37)

**Fig. 5:**
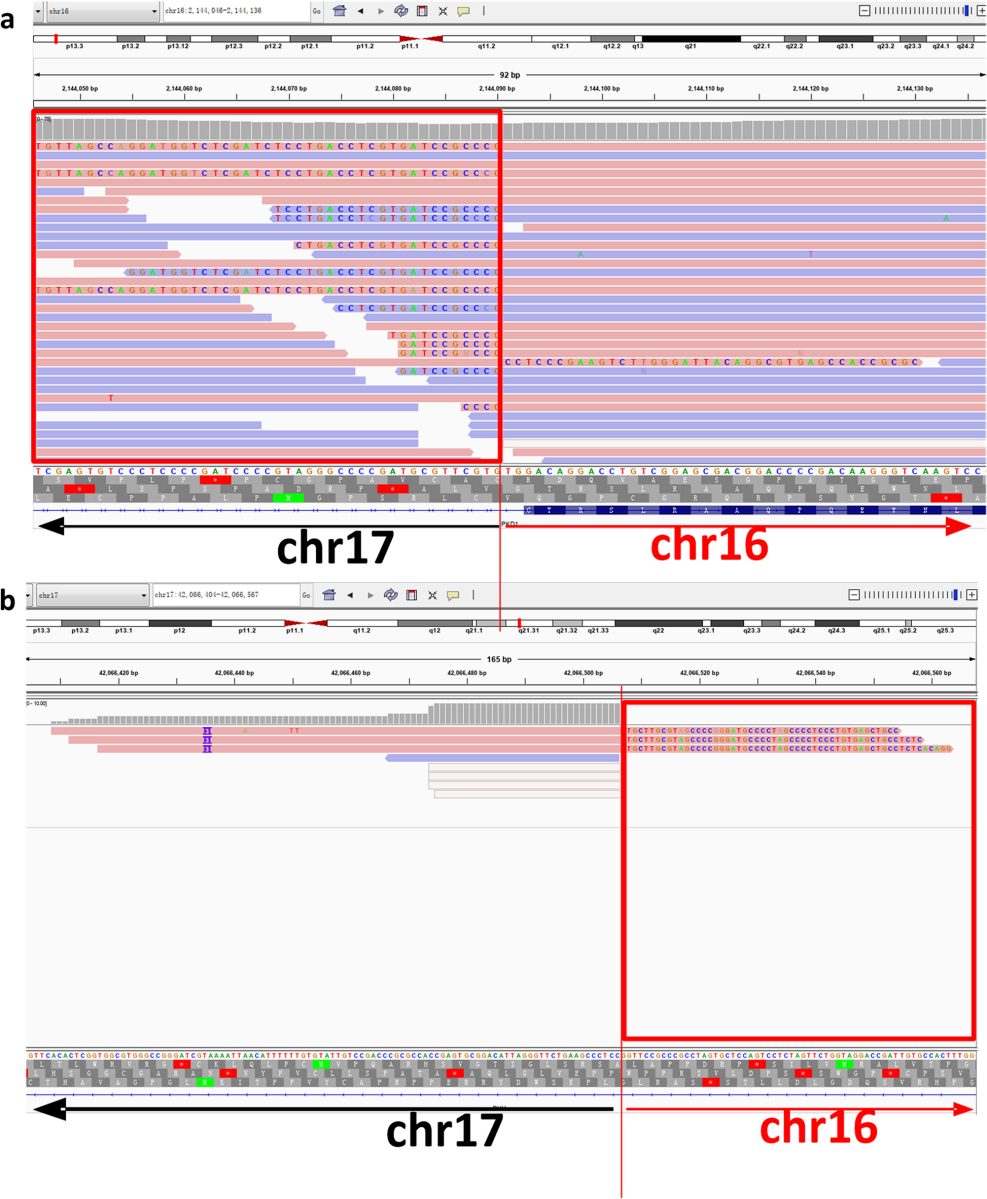
Whole exon sequencing results of patient. **a** Mutation analysis results of the chromosome 16; **b** Mutation analysis results of the chromosome 17

No causal variant was identified by WES, but karyotyping of the patient is 46, XX, t (16; 17) (p13.3; q21.3) (Fig. 3). The translocation locates between 16p13.3 and 17q21.3, and PKD1gene is located in the region of 16p13.3. OGM analysis showed balanced and reciprocal translocation, t(16;17)(p13.3;q21.31), with the breakpoints falling into PKD1 gene intron 14 ~ exon 46, between chr16: 2,138,218-2,161,988, the breakpoints of chr17 were in intron 1 of PYY gene, between chr17: 42,057,369-42,069,730 (Fig. 4). Based on the OGM breakpoints, the WES data close to the breakpoints were revisited and a structure variant covering intron 35 of the PKD1 gene and intron 1 of the PYY gene was determined (Fig. 5).

### Breakpoint validation by sanger sequencing

**Fig. 6:**
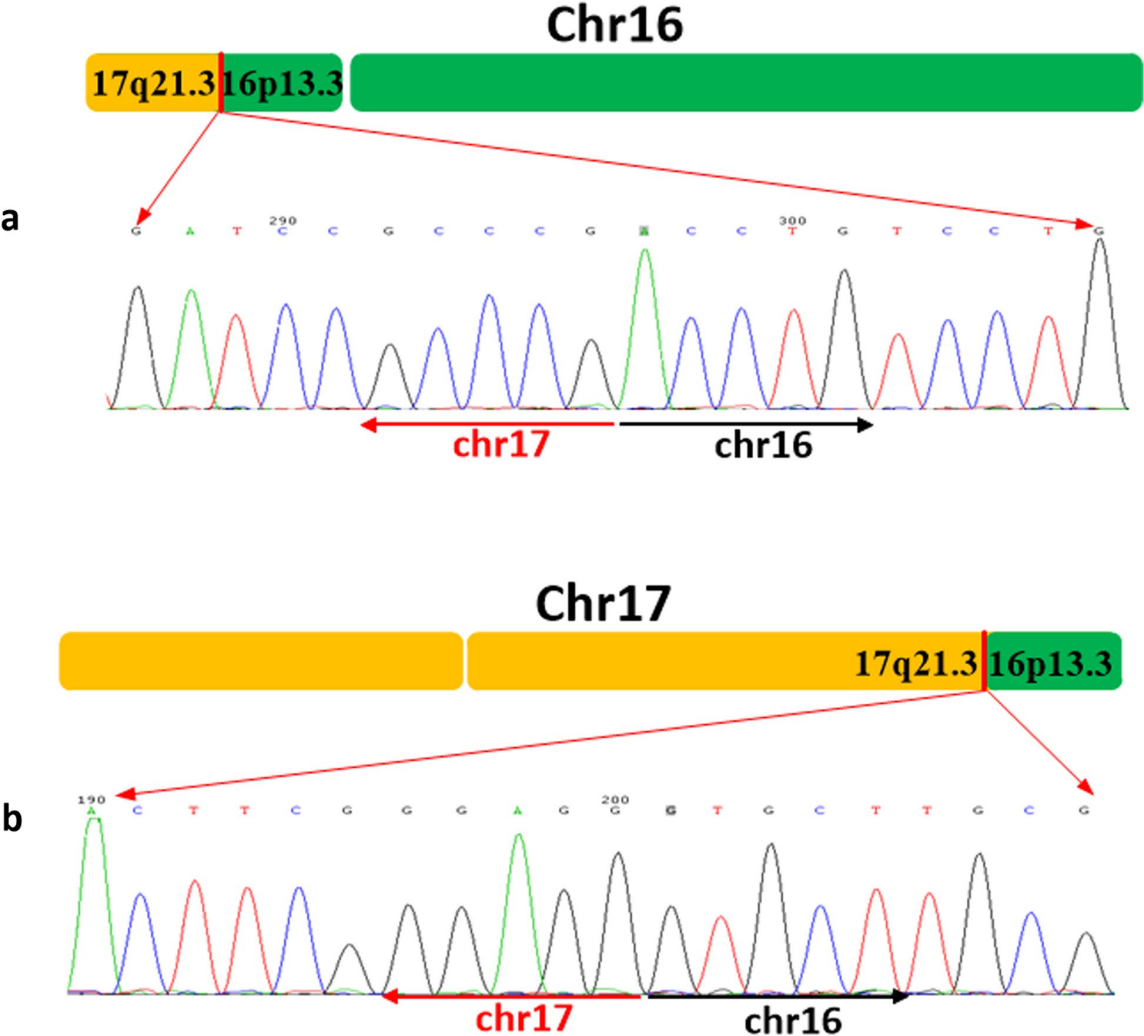
Sanger validation of the breakpoints. **a** Sanger sequence of proband chromosome 16; **b** Sanger sequence of proband chromosome 17

Both PCR and subsequent Sanger sequencing results of the proband showed that c.10618 + 3 (chr16:2144090) of the PKD1 gene is translocated to c.463–15,246 (chr17:42066513) of the PYY gene (Fig. 6). The mother and father of the proband are negative in PCR.

### *PKD1* RNA isolation and reverse transcription-PCR assay

**Fig. 7:**
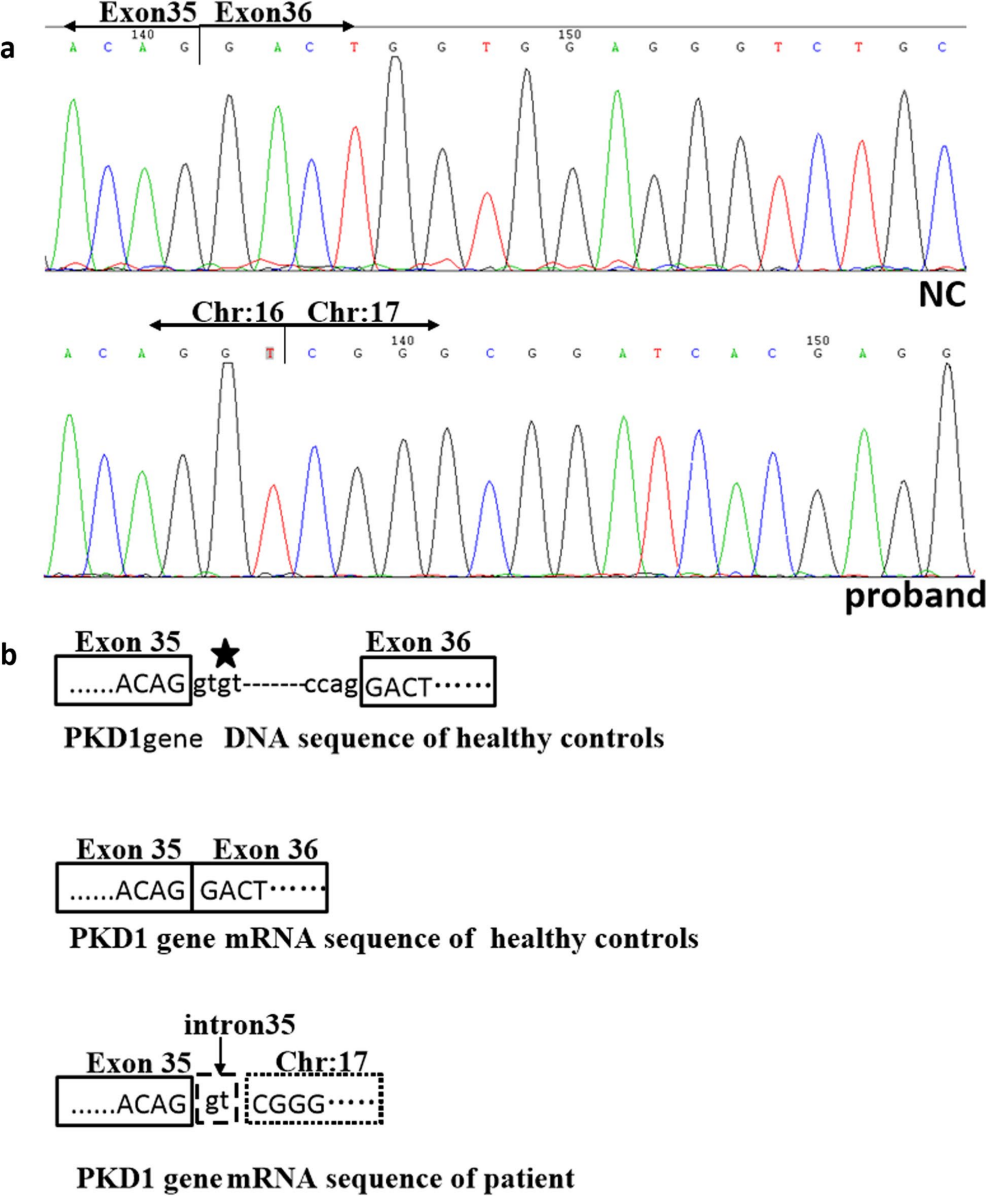
Mutation analysis of PKD1 gene mRNA. **a** Sanger sequencing results of *PKD1* cDNA, the arrow represents cDNA sequence of the healthy control (NC) and the proband. **b** Pattern diagram of mRNA splicing, the asterisk represents the mutant base

The variant of *PKD1* cDNA sequencing results showed that this SV is predicted to lead to a frameshift resulting in a premature STOP codon (p. Pro3541Argfs*17) (Fig. 7). The breakpoint on chromosome 17 (chr17:42066513) locates in intron 1 of the PYY gene (c.-463 + 15,246), whose coding sequence begins in exon 5.

### Genetic counseling

Our patient suffers from the ADPKD caused by balanced translocation, while her parents have normal karyotype. We explained to her that according to the existing theories, she has a 16 in 18 risk of pregnancy failure or miscarriage when reproducing, a 1 in 18 chance of having an affected child (who suffers from ADPKD), and a 1 in 18 chance of having an unaffected child. PGT-SR/M can be employed to determine the chromosomal status of embryos, thereby enable her to plan for a healthy child. Nevertheless, due to technical limitations, PGT-SR/M cannot completely excluded the risk of adverse pregnancy, thus prenatal diagnosis must be performed at the 4th month into pregnancy.

### 3 embryos were obtained for PGT

The couple in this study received 2 PGT treatment cycles, and a total of 3 blastocysts (E1-C1, E1-C2, & E4-C2) were biopsied for WGA.

### One embryo is unbalanced embryo and 2 are balanced after PGT-A

**Fig. 8:**
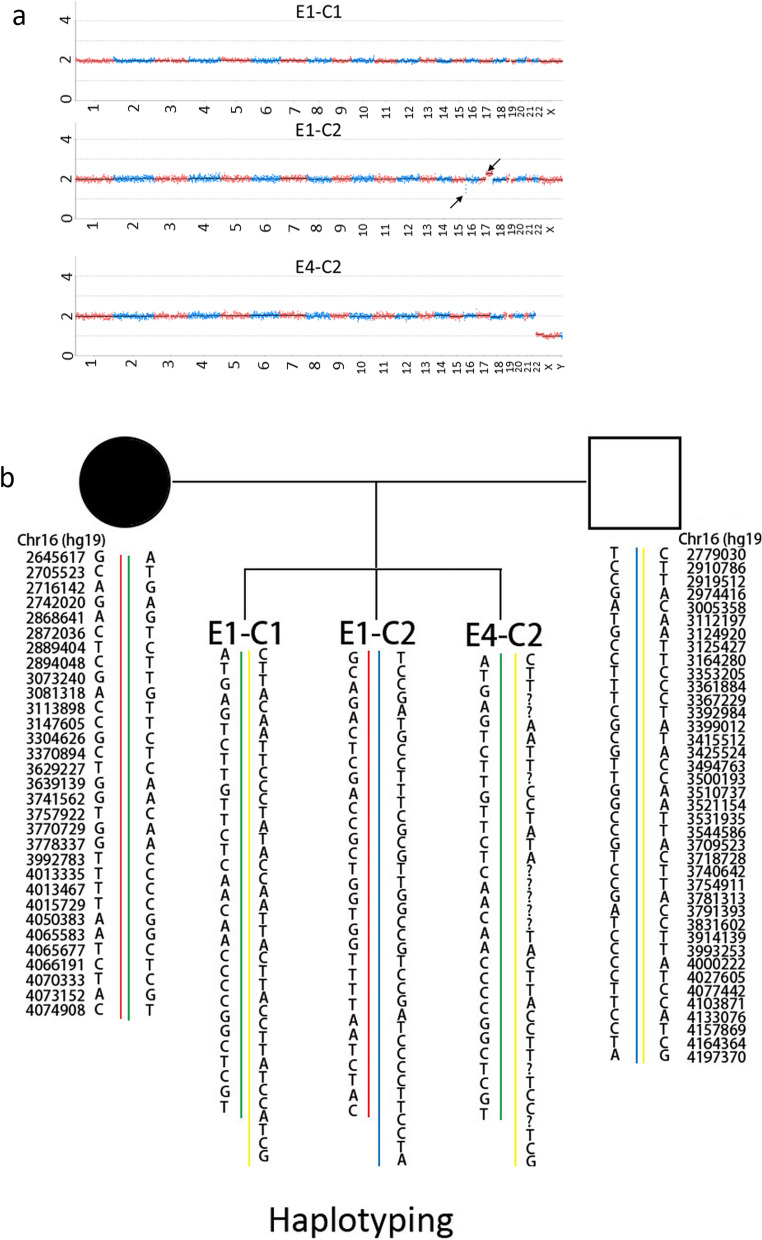
PGT-SR results of the embryos. **a** Chromosome screening results of embryos. The arrow points to abnormal chromosomes; E1-C1, E1-C2, E4-C2: embryos from the patient; **b** PGT-SR results of the pedigree. The red line represents the translocated chromosome 16 of the proband, and the green line represents the normal chromosome 16 of the proband. The normal chromosomes 16 of the husband are shown in blue and yellow

Embryo E1-C2 was identified as an aneuploid embryo, and embryos E1-C1 and E4-C2 were euploidy, indicating that the euploid embryos were either translocation carrier or non-carrier (Fig. 8a). The linkage analysis showed the 2 blastocysts were non-carrier (Fig. 8b).

### Junction-spanning PCR and sanger sequencing results of embryos

**Fig. 9:**
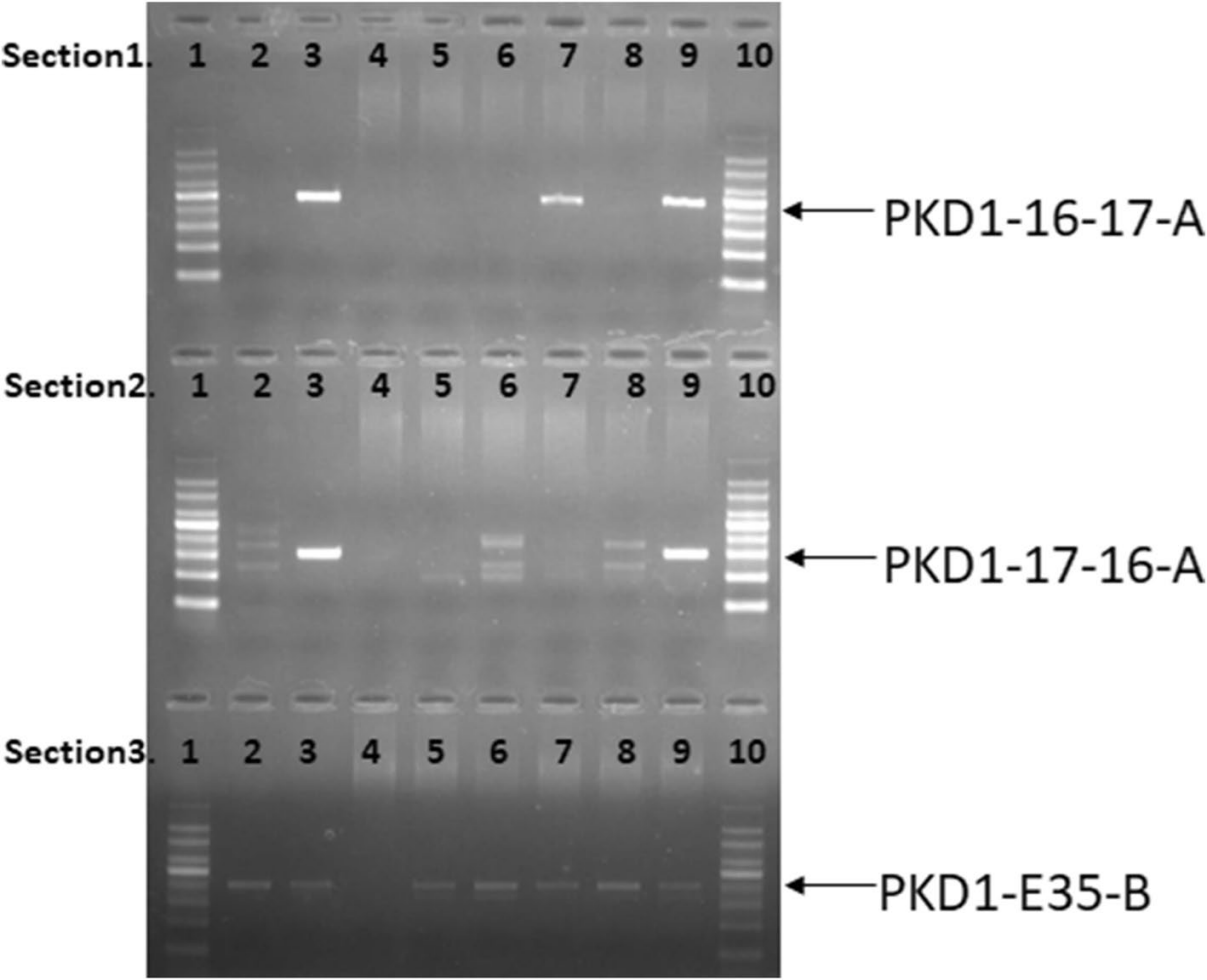
Identification of noncarriers from euploid embryos using PCR. Section.1: PKD1–17-16-A, chromosomal 16 breakpoint region primers; Section.2: PKD1–16-17-A, chromosomal 17 breakpoint region primers; Section.3: PKD1-E35-B, the normal chromosomal 16 primers flanking the breakpoints; Lane 1, 100 bp DNA ladder; lane 2, control peripheral blood genomic DNA; lane 3, peripheral blood genomic DNA of the proband; lane 4, blank control; lane 5, control embryos; lane 6, embryo E1-C1 from the patient; lane 7, embryo E1-C2 from the patient; lane 8, embryo E4-C2 from the patient; lane 9, degenerates embryo; Lane 10, 100 bp DNA ladder

Junction-spanning PCR results showed that Chr16 breakpoint product was amplified and Chr17 breakpoint product was not amplified in one embryo (E1-C2), while the other two embryos (E1-C1 and E4-C2) had normal PCR products (Fig. 9). These results indicated that embryo E1-C2 is an affected aneuploid embryo; and E1-C1and E4-C2 embryos are normal. The junction-spanning PCR results showed 100% agreement with the previous linkage analysis results.

### Prenatal and postnatal testing

Based on the PGT results of the 3 embryos, embryo E4-C2 was chosen and implanted into uterus.

**Fig. 10:**
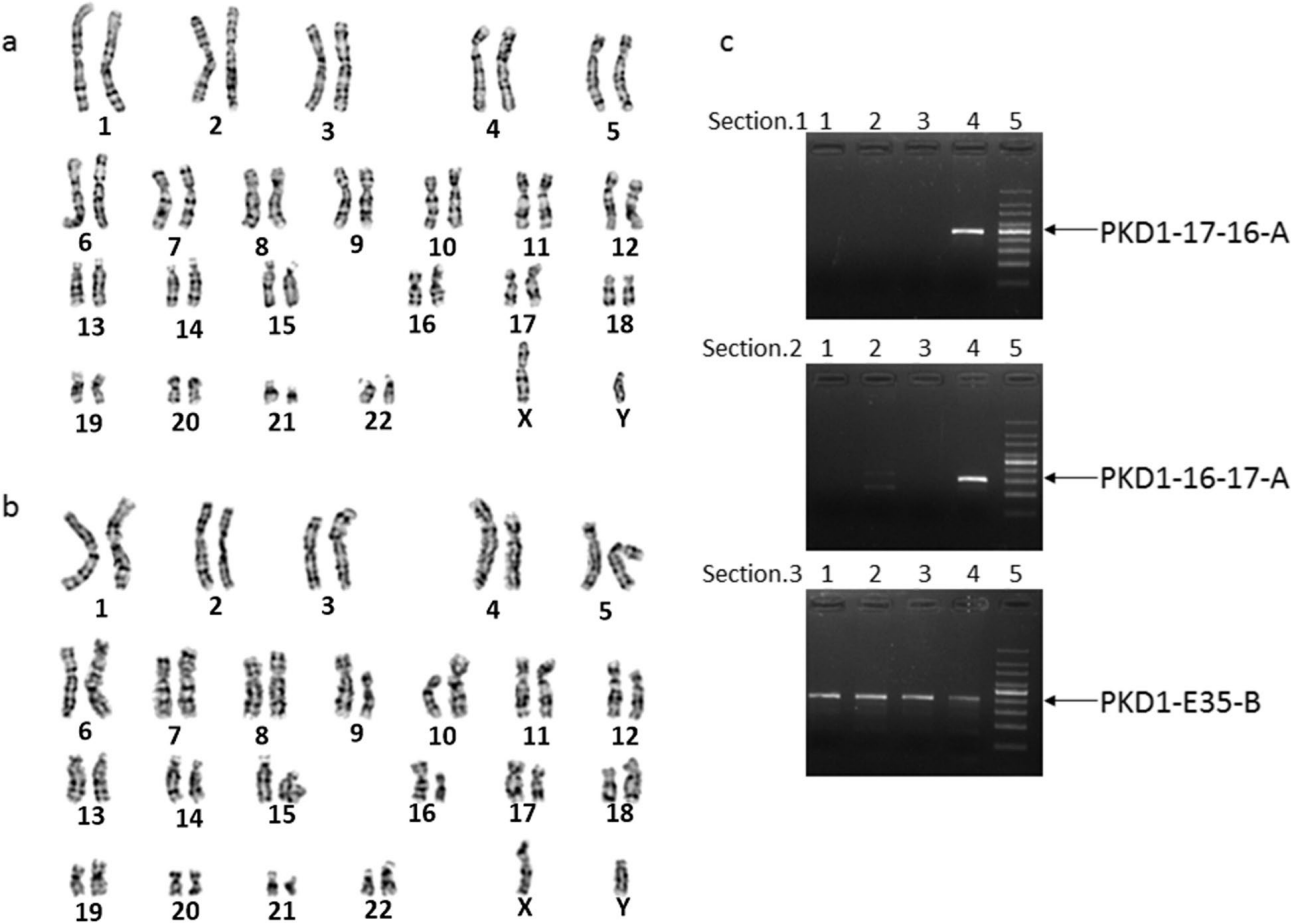
Prenatal and postnatal testing results. **a** G-band karyotype of the fetus. **b** G-band karyotype of the newborn. **c** Gel electrophoresis of the PCR product designed to detect the SVs. Section.1: PKD1–17-16-A, chromosomal 16 breakpoint region primers; Section.2: PKD1–16-17-A, chromosomal 17 breakpoint region primers; Section.3: PKD1-E35-B: the normal chromosomal 16primers flanking the breakpoints; Lane 1, genomic DNA of amniotic fluid; lane 2, genomic DNA of umbilical cord blood; lane 3, control peripheral blood genomic DNA; lane 4, peripheral blood genomic DNA of the proband; Lane 5, 100 bp DNA ladder

Karyotyping of amniotic fluid, collected at 22+ weeks of pregnancy, showed the fetus is 46, XY (Fig. 10). The ultrasonogram showed that the fetal liver and kidneys were developing normally at 28+ weeks of pregnancy. Karyotype of umbilical cord blood at birth showed 46, XY (Fig. 10).

## Discussion

ADPKD is a hereditary disease with complex phenotype and genetic heterogeneity, and most are caused by variants of the *PKD1*(16p13.3, 78%) or *PKD2* (4p21, 15%) gene. The types of variant include SNV(single nucleotide variants) (including missense, splicing, nonsense, small fragment deletion/duplication/insertion, etc.), CNV (including multiple exons and whole gene deletion/duplication) [21] and SVs. So far, SNV accounted for 92.15% (763/828) of pathogenic and likely pathogenic variants in the ClinVar database (https://www.ncbi.nlm.nih.gov/clinvar/). Therefore, WES is mainly used to detect SNV of related genes (*PKD1, PKD2, PKHD1, HNF1B, GANAB, UMOD, NOTCH2, DNAJB11,* etc.) in patients with polycystic kidney disease. However, WES can not provide a complete view of such human genetic variation as SVs. SVs, including repeat expansions, insertions, deletions, or rearrangements, probably are responsible for many undetected disease-causing variants, but existing short-read sequencing technologies often fail to identify them.

In this study, a proband was diagnosed with polycystic kidney disease during a routine physical examination and she had a history of two miscarriages. Routine WES analysis result was negative, but a chromosomal translocation between chr16 and chr17 was found by karyotyping. Then OGM found concordant t(16;17)(p13.3;q21.31), and the breakpoints overlapping PKD1 gene, producing a truncated PKD1 protein(Polycystin-1, PC1) without complete transmembrane and C-terminal domains, and subsequently leading to physical linkage broken between PC1 and PC2(Polycystin-2)). Meanwhile, the breakpoint on chromosome 17 (chr17:42066513) locates in intron 1 of the *PYY* gene (c.-463 + 15,246), whose coding sequence begins in exon 5. The PYY gene encodes the peptide YY, which acts on the gastrointestinal tract as an inhibitor of gastric acid secretion, gastric emptying, digestive enzyme secretion by the pancreas, or gut motility. The obesity susceptibility in PYY gene has not been confirmed in Online Mendelian Inheritance in Man (OMIM). No validated pathogenic variants were recorded in the Human Genetic Mutation Database (HGMD) or ClinVar until February 13, 2022. This patient’s BMI was 23.4 kg/m^2^, which was normal.

Reciprocal translocation is common in the human population, and mainly produced by two mechanisms, namely non-homologous end joining (NHEJ) and non-allelic homologous recombination (NAHR). In this study, there was no high proportion of homologous region or clear palindromic sequence near the breakpoint, so it is presumed that the equilibrium translocation is a non-repetitive rearrangement generated by non-homologous end junction (NHEJ). In the conventional detection methods, NGS-based WES or CNV-seq cannot effectively detect translocation due to the limited read length. G-banded karyotype is still an economical and effective method, yet it can’t identify either the breakpoints or the affecting genes. With the development of technologies, the specific breakpoint of translocation has been located at base level by long-read sequencing, such as Pacbio, Nanopore [22, 23]. However, long read-sequencing technologies also generate high assembly error rates. OGM, on the other hand, is able to detect all types of structural variants (SVs) with identifying breakpoints at kilobasepair resolution. It enables a more accurate determination of structural rearrangements such as translocation breakpoints [24, 25]. For those monogenic diseases caused by breakpoint translocation inside the pathogenic genes, it is crucial to determine the breakpoint position, especially in PGT and prenatal diagnosis. Nanopore and other technologies have been applied in clinical PGT, providing more options for patients with reciprocal balanced translocation [22].

Chromosomal structural abnormalities, also known as structure rearrangement (SR), especially reciprocal translocations, have been documented to cause a variety of phenotypes, including infertility, disease syndromes, and congenital abnormalities. In the past two decades, PGT-A has helped couples with translocation select euploid embryos for transference and then deliver offsprings with normal phenotype. This strategy can grant them their own babies, but the babies are translocation carriers sometimes and very likely to encounter similar fertility problems when grow up. In recent 5 years, long-read sequencing methods were involved in preimplantation genetic testing for structural rearrangements (PGT-SR) and the euploid translocation carrier embryos were selected out as a backup, but not a priority for transferring, because a non-carrier embryo is not guaranteed in any PGT cycle, and a carrier embryo can also be transferred if no additional genetic risk is detected. For autosomal dominant genetic diseases caused by SR, the carrier embryo is at genetic risk, so it is not a recommended choice for implantation. In this study, the patient suffered a polycystic kidney phenotype and two miscarriages due to PKD1 gene disruption caused by reciprocal translocation. PGT intervention can usually be fulfilled in two ways: PGT-M and PGT-SR. In our case, when using PGT-SR strategy, it’s a necessity to select non-carrier embryos for transferring so as to avoid the genetic defects at birth.

## Conclusions

Our study shows that monogenic genetic diseases caused by structural rearrangement may not be detected by NGS. The genetic diagnosis and subsequent assisted reproductive intervention can be done through the following procedure: firstly, combination of karyotype, OGM and Sanger sequencing can be performed to increase the diagnostic yield; secondly, finding the breakpoints of monogenic genetic disorders caused by reciprocal translocations is important for accurately assessing the genetic risk and giving appropriate genetic counseling. This gives patients more options when it comes to having the offspring. For example, the patient in this study could choose PGT-SR/M or prenatal diagnosis; thirdly, when performing PGT-SR for autosomal dominant genetic diseases caused by reciprocal translocations, non-carrier embryos should be selected for implantation in order to avoid birth defects.

## Data Availability

The datasets generated and analyzed during the current study are available in the [GenBank] repository (GenBank (https://www.ncbi.nlm.nih.gov/WebSub/) ID: 2266173). Other data used for the analyses of this study are available from the corresponding authors upon reasonable request.

## Abbreviations

WES: Whole exome sequencing
ADPKD: Autosomal dominant polycystic kidney disease
OGM: Optical Genome Mapping
CMA: Chromosome microarray
HMW: High molecular weight
NGS: Next generation sequencing
IVF: In vitro fertilization
PGT: Preimplantation genetic testing
PGT-SR: Preimplantation genetic testing for structural rearrangements
PGT-A: Preimplantation Genetic Testing for aneuploidy
PGT-M: Preimplantation Genetic Testing for monogenic conditions
SVs: Structure variantions
SNV: Single nucleotide variants
CNV: Copy number variation
OMIM: Online Mendelian Inheritance in Man
HGMD: Human Genetic Mutation Database
NHEJ: Non-homologous end joining
NAHR: Non-allelic homologous recombination
